# The Spectrum of Headache in Leptomeningeal Metastases: A Comprehensive Review with Clinical Management Guidelines

**DOI:** 10.1007/s11916-023-01180-9

**Published:** 2023-10-24

**Authors:** Jessica A. Wilcox, Rachel Estrera, Adrienne Boire

**Affiliations:** 1 Department of Neurology, Memorial Sloan Kettering Cancer Center, New York, NY USA; 2 Human Oncology and Pathogenesis Program, Brain Tumor Center, Memorial Sloan Kettering Cancer Center, New York, NY USA

**Keywords:** Headache, Leptomeningeal metastases, Intracranial pressure, Intrathecal chemotherapy, Whole-brain radiation therapy

## Abstract

**Purpose of Review:**

Headaches are a common, oftentimes debilitating symptom in patients with leptomeningeal metastases.

**Recent Findings:**

The third edition of the International Classification of Headache Disorders provides a useful diagnostic framework for headaches secondary to leptomeningeal metastases based on the temporal relationship of headache with disease onset, change in headache severity in correlation with leptomeningeal disease burden, and accompanying neurologic signs such as cranial nerve palsies and encephalopathy. However, headaches in patients with leptomeningeal metastases can be further defined by a wide range of varying cancer- and treatment-related pathophysiologies, each requiring a tailored approach.

**Summary:**

A thorough review of the literature and expert opinion on five observed headache sub-classifications in patients with leptomeningeal metastases is provided, with attention to necessary diagnostic testing, recommended first-line treatments, and prevention strategies.

## Introduction

Leptomeningeal metastases are defined as the infiltration of cancer cells into the cerebrospinal fluid (CSF) and are regarded as an incurable stage of advanced cancer. An estimated 5–25% of patients with solid tumor and hematologic malignancies will develop leptomeningeal metastases throughout their disease course, most commonly affecting those with metastatic lung cancer (9–25%), breast cancer (5–20%), melanoma (6–18%), and non-Hodgkin’s lymphoma (6–8%) [1, 2, 3•, 4–7]. A complex and highly dynamic process, leptomeningeal metastases disseminate along the neuraxis in two distinct states: (1) floating cells within the subarachnoid space detectable by CSF cytologic examination, and (2) adherent plaques to the surface of the brain, spinal cord, and exiting cranial and peripheral nerves that are visible on contrast-enhanced magnetic resonance imaging (MRI) [8, 9]. Patients with leptomeningeal metastases experience a high burden of rapidly progressive neurologic signs and symptoms, including head and back pain, cranial nerve palsies, gait instability, bowel and bladder dysfunction, and cognitive changes [10].

**Fig. 1:**
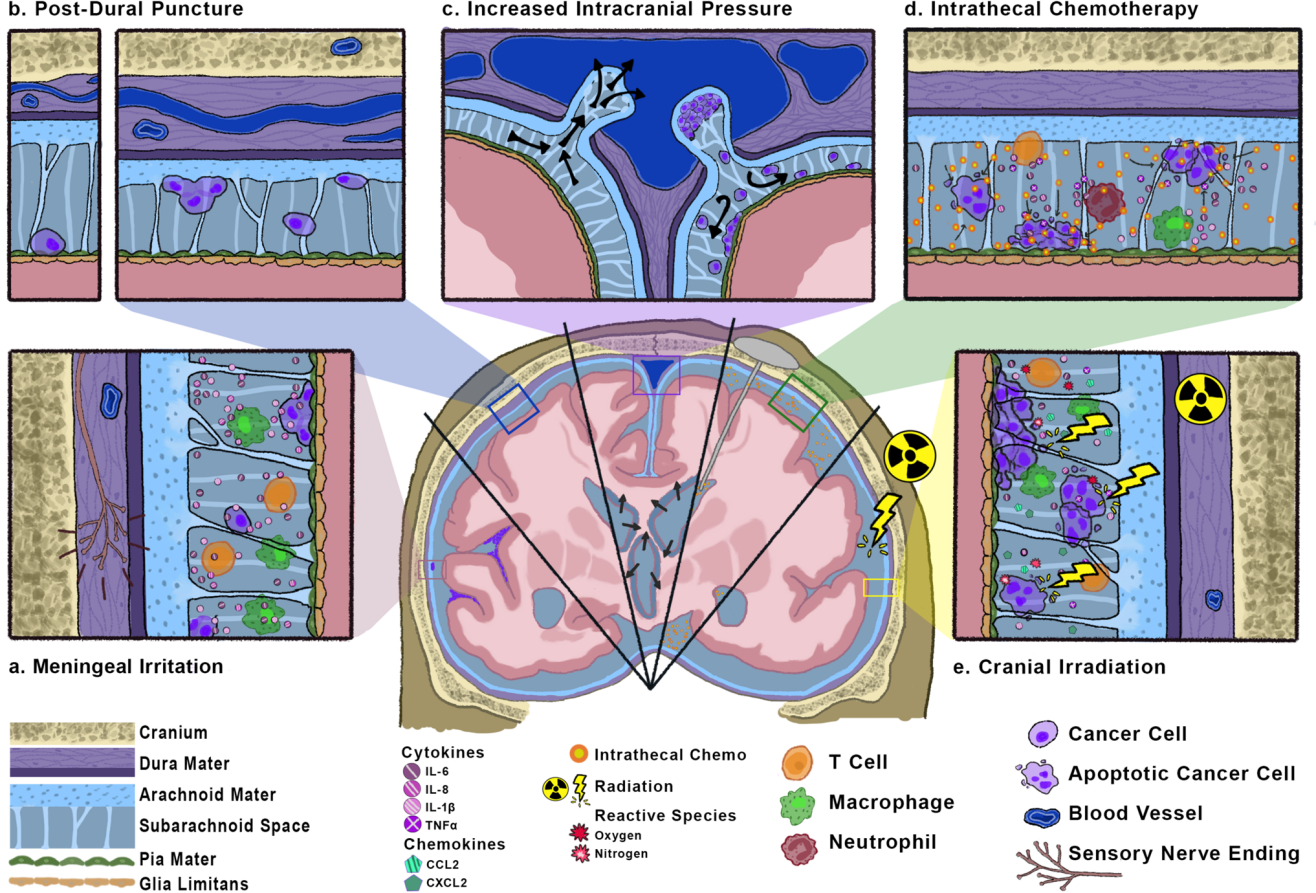
Pathophysiologic representation of five headache subtypes in patients with leptomeningeal metastases. In the setting of leptomeningeal metastases, cancer cells can be found free-floating in the CSF or adherent to the leptomeninges, transitioning between the adherent and floating states. **a** In disease-related meningeal irritation, sensory nerves embedded in the dura mater are theorized to experience innate-mediated activation. Myeloid cells (e.g., macrophage) and lymphoid cells (e.g., T cells) outnumber cancer cells, and higher levels of inflammatory cytokines (IL-6, IL-8, IL-1β) are observed. **b** Post-dural puncture headaches result from persistent CSF leakage incident to the dural hole and intracranial hypotension. On contrast-enhanced MRI brain, pachymeningeal thickening and enhancement are observed; a proposed explanation is compensatory dural vein engorgement. **c** Elevated intracranial pressure arises as cancer cells fill arachnoid granulations, disrupting the normal drainage of CSF from the subarachnoid space into dural venous sinuses. Brain imaging reveals communicating hydrocephalus or “ballooning” of the ventricles. **d** Intrathecal chemotherapy delivered via Ommaya reservoir causes a chemical arachnoiditis in the subarachnoid space, marked by increased leukocytes (T cells and macrophages), granulocytes (neutrophils), and inflammatory cytokines. **e** Ionizing radiation activates resident microglia, releasing pro-inflammatory cytokines (IL-6, IL-8, IL-1β, TNFα), chemokines (CCL2, CXCL2), and reactive oxygen and nitrogen species into the CSF. With increased blood-brain and blood-CSF barrier permeability, additional T lymphocytes and macrophages infiltrate the subarachnoid space and add to the inflammatory milieu

Headache is common in patients with leptomeningeal metastases. The incidence of headaches is estimated at 32% of patients at initial diagnosis and up to 75% throughout their disease course [11, 12]. The International Headache Society’s third edition of the International Classification of Headache Disorders (ICHD-3) defines “headache attributed to carcinomatosis meningitis” as requiring two of the three criteria in a patient with known leptomeningeal metastases: Headache has developed in temporal relationship to leptomeningeal metastases, headache has either significantly improved in the setting of improving leptomeningeal metastases or significantly worsened in the setting of progressive leptomeningeal metastases, and headache is associated with cranial nerve palsies and/or encephalopathy [13]. While this definition provides a useful framework, patients with leptomeningeal metastases can have several, sometimes overlapping, headache subtypes with distinct differences in pathophysiology (Fig. 1), presentation, and clinical management.

## Leptomeningeal Neuroanatomy

Before detailing the various headache phenotypes observed in patients with neoplastic meningitis, an understanding of the anatomic makeup of the meninges and normal composition and function of the CSF is essential to conceptualize dynamic changes associated with leptomeningeal metastases.

### Anatomy of the Dural Meninges

The cerebral meninges are composed of three layers: dura, arachnoid, and pia mater. The outermost dura mater encasing the brain and spinal canal is a thick, fibrous tissue that is composed of periosteal (skull-facing) and meningeal (arachnoid-facing) membranes, largely fused with the exception of intervening intracranial dural venous sinuses [14]. The intracranial dura receives a rich vascular supply from branches of the internal and external carotid and vertebral arteries, and meningeal veins traverse the periosteal layer before communicating with veins of the skull and cerebrum to drain into dural sinuses [14]. Sensory innervation to the cranial dura is largely supplied by the trigeminal nerve, the first three cervical cranial nerves, and the cranial sympathetic trunk, with additional sensory branches from the facial, vagus, glossopharyngeal, and hypoglossal nerves [14]. Any inflammatory or painful insult within the cranium, including leptomeningeal metastases, causes headache by activation of the pain fibers within the dura mater [15].

### Anatomy of the Leptomeninges

Below the dura mater, the arachnoid mater and pia mater comprise the leptomeninges. The arachnoid layer is adherent to the dura mater and bridges cerebral sulci, whereas the pia mater follows the contours of cranial sulci and envelopes the spinal cord. The arachnoid mater is avascular, composed of both a dorsal subdural layer and a ventral pial-facing layer, with intervening trabeculae of collagen bundles that traverse the CSF-containing subarachnoid space [14]. The pia mater comprises an epipial layer of collagen fibers contiguous with arachnoid trabeculae and a deeper intima layer composed of elastic and reticular fibers that anchor to the neural tissue by a glial membrane. The pia contains a rich capillary network and invaginates to form peri-vascular sheaths around blood vessels entering and exiting the brain parenchyma.

### CSF Macroenvironment

The majority of CSF is produced by the choroid plexi, highly vascular structures composed of a fenestrated capillary network within a loose connective stroma and encased in a specialized choroidal epithelium [16]. Tight junctions between choroid epithelial cells and between astrocytes within the basement membrane of the pia mater form the blood-CSF barrier, a distinct entity from the blood-brain barrier [17]. The ventricles and subarachnoid space contain approximately 150 mL of CSF in a healthy adult [4, 18, 19]. CSF flows in a pulsatile cranio-caudal manner from the lateral ventricles through the foramen of Monro into the third ventricle, through the cerebral aqueduct into the fourth ventricle, and then through the medial foramen of Magendie and lateral foramen of Luschka to the subarachnoid spaces at the base of the brain, cerebral convexities, and spinal cord. CSF then drains into the venous circulation primarily via arachnoid granulations, which are outpouchings of the arachnoid mater scattered along the dural venous sinuses and by lymphatic channels [20–22]. CSF is produced on a continuous basis at a rate of 20 mL/h and turns over approximately 2–3 times throughout the course of a single day.

### CSF Microenvironment

CSF maintains the brain and spinal cord in a floating suspension, providing multiple critical functions to the central nervous system (CNS). CSF protects the brain from external mechanical forces as a shock absorber and provides buoyancy to the brain in order to limit weight and traction on the base of the skull. The total volume of CSF fluctuates in response to changes in intracranial blood volume in order to maintain adequate cerebral perfusion pressure [23]. CSF also serves as a conduit for distribution of neuroendocrine factors and clearance of endogenous metabolic waste. The CSF contains a sparse amount of micronutrients, ions, peptides, and proteins, either secreted from the blood or synthesized in the choroid plexus, that are essential for healthy brain functioning [24]. While paucicellular and classically believed to represent an “immune-privileged” site, CSF under healthy conditions is home to a low number of meningeal macrophages and trafficking memory T cells, with a much smaller proportion of B cells, monocytes, and dendritic cells [25•]. Myeloid cells within the choroid plexus and meninges serve critical antigen-presenting functions to circulating memory T cells in the subarachnoid space, forming the basis for inflammatory and autoimmune conditions.

## Headache Sub-classifications in Leptomeningeal Metastases

### Headache Secondary to Disease-Related Meningeal Irritation

#### Pathophysiology

The most common type of headache afflicting patients with leptomeningeal metastases, particularly early in the disease course, is generally attributed to meningeal irritation secondary to tumor cell and immunoinflammatory infiltrate within the subarachnoid space. Leptomeningeal cancer cells are commonly outnumbered by a large influx of both myeloid (macrophage, dendritic cells, neutrophils) and lymphoid (T cells, B cells, NK cells) cell types, hence the historical term “carcinomatosis meningitis” [26•]. A rise in TGFβ1-mediated pathways and several inflammatory cytokines (IL-6, IL-8, IL-1β) are observed in the CSF of patients with leptomeningeal metastases, influencing pathways involved with innate immunity and complement activation, acute phase reactions, cellular adhesion and chemotaxis, proteosome activation and inhibition, and tissue damage and repair [26•, 27]. The same inflammatory cytokines have been implicated in the pathogenesis of migraine headaches, which are also theorized to result from innate-mediated activation of the trigeminal nocireceptors in the dura mater, though this mechanism in migraine has been debated [28–30]. Inflammatory signaling pathways tend to positively correlate with leptomeningeal cancer progression, with higher inflammatory signaling in those with poor response to cancer-directed therapy [27]. One explanation for this observation is that despite robust inflammatory signaling, cytotoxic responses are blunted due to a shift towards immunosuppressive cell phenotypes [32]. A higher number of exhausted and inactivated T lymphocytes have been discovered the CSF of patients with leptomeningeal metastases compared to the immune microenvironment of parenchymal brain metastases [27–31].

#### Presentation

Headaches secondary to meningeal irritation tend to be intermittent, dull or stabbing in character, and mild-to-moderate in intensity. The meningitic headache may vary in location within an individual patient, alternating between focal, holocephalic, or cervicogenic. Mild accompanying nausea and photophobia may be present. Classical, meningitic neck stiffness is only present in 15% of patients, resulting from noxious stretch to the inflamed cervical meninges [11].

#### Diagnosis and Management

Diagnosis of meningeal headache rests primarily on the clinical history, with new onset headache or worsening of pre-existing headaches in the setting of leptomeningeal metastases without red-flag signs to suggest other potential causes. Meningeal inflammation from neoplastic causes tends to be less robust than that seen with bacterial or viral infections, and therefore, the classic meningeal signs including Kernig’s or Brudzinski’s sign are generally absent in carcinomatous meningitis [32]. However, diagnostic studies are important to exclude other more serious headaches subtypes that may co-exist in this patient population. On contrast-enhanced MRI, a focal nodular region of leptomeningeal enhancement or lack of T2/FLAIR suppression may correlate with headache location, theoretically due to localized inflammation and regional activation of nocireceptors within the overlying dura [33]. The CSF of patients with leptomeningeal metastases may demonstrate a sterile myeloid or lymphocytic pleocytosis (median 33 cells/µL) and elevated protein levels (median 109 mg/dL) in approximately 80% of patients, but neither is required to fulfill the diagnosis of meningeal irritation [34, 35]. Intracranial pressure (ICP) measurements via manometric reading should be normal, which helps to distinguish meningeal headaches from pain secondary to increased ICP.

Meningeal headaches respond well to corticosteroids (i.e., dexamethasone), non-steroidal anti-inflammatory drugs (NSAIDs), and acetaminophen. However, in advanced disease, meningeal headaches can become refractory to these measures.

#### Prevention

Meningeal headaches may be present with fluctuating severity throughout the disease course of leptomeningeal metastases. While anti-inflammatory agents are highly effective in treating these headaches, long-term use of these agents is wrought with deleterious side effects, e.g., steroid myopathy and weight gain due to steroids and gastritis due to NSAIDs. Since headaches in patients with leptomeningeal metastases tend to be multifactorial, a trial of a prophylactic headache agent used in other primary headache subtypes, such as migraine or tension-type headache, is reasonable to pursue. Agent of choice can be tailored to the individual scenario, for example, amitriptyline in a patient with insomnia or cancer-related dysthymia, gabapentin in a patient with osseous or cauda equina disease with radicular leg pain, and topiramate in a patient with modestly elevated ICP not yet requiring operative CSF diversion. The safety and efficacy of injectable preventative treatments for migraine, such as botulinum toxin and calcitonin gene-related peptide inhibitors, are understudied in patients with leptomeningeal metastases with migrainous overlay, but may in theory offer more rapid therapeutic relief than oral prophylactic agents [36, 37].

### Headache Secondary to Increased Intracranial Pressure

#### Pathophysiology

A second critically important headache subtype in patients harboring leptomeningeal metastases is headache secondary to increased ICP, present in at least 26% of patients [38]. Under normal conditions, CSF exists in a state of equilibrium between production by the choroid plexus, circulation throughout the subarachnoid space, and absorption by arachnoid granulations into the dural venous sinuses. This continuous secretion and absorption maintain a normal ICP of 10–20 cm H_2_O. Disruption of this equilibrium via cancer cell interruption of arachnoid granulation function and normal drainage pathways is thought to impair normal absorption of CSF into the venous circulation, resulting in steadily increased ICP and secondarily headaches.

While headache and accompanying symptoms from increased ICP tend to be nearly continuous, a unique phenomenon referred to as “pressure” or “plateau” waves due to paroxysmal elevations in ICP > 50 mmHg lasting for 5–20 min can also be observed [39, 40]. Plateau waves are most commonly observed in leptomeningeal metastasis patients following a rapid position change, whereby autoregulatory cerebral vessel dilatation and an acute rise in cerebral blood volume result in a rapid rise in ICP and consequently a reduction in cerebral perfusion pressure [41]. Clinically, this manifests in sudden-onset headaches with accompanying emesis, impaired consciousness, and focal neurologic deficits such as lateralizing weakness or aphasia. Therefore, pressure waves may be easily confused with a stroke or seizure and a high index of suspicion is critical in appropriately assessing such patients.

#### Presentation

The International Headache Society defines headache attributed to ICP as increased CSF pressure > 25 cm CSF measured by lumbar puncture in the lateral decubitus position without sedatives or by epidural/ventricular monitoring, with evidence of causality by temporal relationship in headache to intracranial hypertension or by headache relief by reduction in ICP [13]. Headaches secondary to increased ICP are classically holocephalic, throbbing in quality, moderate-to-severe in intensity, and worse in the setting of supine positioning, cough, or sudden position changes. Pressure-mediated headaches often awaken the patient from sleep, theorized to result from gravitational changes to the compartmental pressure gradient and also physiologic hypercapnia-induced cerebral vasodilation [42••]. Neck or bi-occipital pain radiating into the shoulders is an alternative, more atypical, headache descriptor. Nausea and vomiting, specifically “unprovoked” emesis, are very common accompanying symptoms, though patients may also describe sudden vomiting in the absence of underlying headache. Patients may experience binocular diplopia secondary to pressure-mediated traction on cranial nerve VI and dimming or blurred vision due to pressure on the optic nerve. Hydrocephalus with stretch imposed on the descending corticospinal tract fibers adjacent to the lateral ventricles can cause imbalance. Mental status changes with progressive confusion, obtundation, and loss of consciousness may also be seen in acute onset or severely elevated ICP.

Neurologic examination findings suggestive of increased ICP include new onset cranial nerve VI palsy, papilledema, wide-based unsteady gait with retropulsion, and obtundation with a mixed or global aphasia characterized by perseverative speech and/or inability follow commands. Vital signs may demonstrate a Cushing triad (bradycardia, hypertension, irregular respirations) in the setting of severe ICP elevations.

#### Diagnosis and Management

Increased ICP is a neurologic emergency and must be handled swiftly to prevent rapid neurologic decompensation. Any patient suspected of increased ICP should undergo a STAT noncontrast head CT to investigate for communicating hydrocephalus and increased transependymal flow in the frontal and occipital horns. In the interpretation of these films, it is wise to remember that acute elevations in ICP do not necessarily induce significant changes in ventricular caliber, particularly in younger patients with non-compliant ventricles [43]. The pace of developing communicating hydrocephalus is critically important; an acute 3 mm increase in third ventricular caliber in a 1-month interval is generally more alarming than a gradual 3-mm increase over the course of 1 year.

Until sufficient CSF diversion is achieved, patients should be maintained with head of bed at 30° and avoid prolonged supine positioning. Common pharmacologic agents for vasogenic edema, such as high-dose dexamethasone, mannitol, and hypertonic saline, play a little role in the treatment of acute hydrocephalus where the indicated treatment is CSF diversion. Medications which gradually reduce CSF production, such as acetazolamide and topiramate, are also generally insufficient to temporize symptomatology or rapidly rising pressures.

Urgent lumbar puncture is both diagnostic and therapeutic. Accurate opening pressure measurement requires the patient to be placed in the lateral decubitus positioning with legs extended, with manometer readings > 20–25 cm H_2_O confirmatory of elevated ICP. Large volume CSF removal of 20–30 cm^3^ often provides rapid but transient improvement in headache and neurologic symptoms, as CSF re-accumulation generally occurs over the subsequent 12–24 h. A more permanent solution involves neurosurgical consultation for placement of a ventriculoperitoneal or ventriculoatrial shunt, which will drain excess CSF into the peritoneal cavity or systemic circulation, respectively. Extracranial shunting in patients with leptomeningeal metastases is a palliative procedure and may allow patients to continue with tumor-directed therapy [44]. However, as the presence of increased ICP is usually indicative of more advanced leptomeningeal metastases and high neurologic symptom burden, survival after extracranial shunt placement is generally only 3–5 months. If within a patient’s goals of care, shunting should be considered in all patients with symptomatic elevations in ICP, with symptom improvement or resolution in approximately 80% of patients [44, 45]. In patients with asymptomatic modest elevations in ICP, a period of close observation may be considered however with a low threshold to consider surgical intervention given risk of rapid neurologic decompensation, particularly in those soon to undergo cranial irradiation.

As headaches in patients with leptomeningeal metastases are quite common, and increased ICP signs may be subtle particularly to the untrained examiner, we recommend lumbar puncture to confirm opening pressure in any patient with new or escalating headaches or refractory, unexplained nausea, or emesis, barring any contraindications to this procedure [46]. Lumbar puncture requires a platelet count > 50 k, INR > 1.5 or institutional standard, appropriate hold of anticoagulation or certain anti-platelet agents, and no evidence of obstructive non-communicating hydrocephalus secondary to bulky periventricular or posterior fossa disease.

#### Prevention

Since elevated ICP is often a sign of a high burden of leptomeningeal metastases, the best preventative strategy against elevated ICP is prompt treatment of the patient’s underlying disease. Early diagnosis of leptomeningeal metastases requires a high level of suspicion. Expedited workup for a patient demonstrating subtle signs of leptomeningeal metastases, including neuraxial gadolinium-enhanced MRI and lumbar puncture, might allow for earlier initiation of CNS-targeted or CSF-penetrant systemic therapies [47]. Leptomeningeal enhancement may be absent in patients exposed to anti-angiogenic agents such as bevacizumab; careful review for subtle radiologic signs indicative of occult leptomeningeal disease including lack of sulcal T2/FLAIR suppression and/or small changes in ventricular caliber is wise. Emerging CSF biomarkers, such as circulating tumor cells and cell-free DNA, aim to improve diagnostic sensitivity of leptomeningeal metastases and facilitate earlier initiation of treatment [48, 49].

### Headache Secondary to Dural Puncture

#### Pathophysiology

In addition to the more commonly observed high ICP headache, patients with leptomeningeal metastases undergoing diagnostic lumbar punctures may also suffer from iatrogenic low ICP and orthostatic headaches. The dural breach created by the LP needle generally heals within minutes to hours following removal of the spinal needle. For this reason, a brief period of supine positioning is often recommended post-procedurally to reduce intensity of a post-dural puncture headache (PDPH), despite lack of evidence for this intervention [50]. However, in 4–40% of patients, persistent CSF leakage at the dural puncture site results in symptomatic intracranial hypotension [51, 52]. Multiple mechanisms have been postulated to explain orthostatic headaches secondary to intracranial hypotension including sagging of intracranial structures and subsequent traction on cranial and cervical sensory nerves, compensatory dilation of venous sinuses and tributary veins following reduction in CSF volume, and alteration of craniospinal elasticity causing increased lumbar relative to intracranial hydrostatic compliance [53, 54]. Risk factors for PDPH include female gender, younger age (18 to 50 years), cutting-type and large bore spinal needles, numerous pass attempts, failure to reinsert the stylet before spinal needle removal, and prior history of PDPH [55, 56].

#### Presentation

The International Headache Society defines PDPH as any headache occurring within 5 days of dural puncture and is typically accompanied by neck stiffness and/or subjective hearing complaints [13]. Patients often describe postural bifrontal or bioccipital headaches, exacerbated with sitting upright or ambulation and improved with supine positioning. Postural nausea and vomiting may also be present. Atypical features within this time frame, such as “worst headache of life,” fevers, focal neurologic symptoms, or confusion, should prompt investigate for potential rare complications of LP including subdural hematoma or infectious meningitis.

#### Diagnosis and Management

Clinical history is often sufficient to diagnosis PDPH, as the postural headache and temporal relationship with lumbar puncture is pathognomonic. Unless alternative etiologies are being considered due atypical features, confirmatory imaging is generally not necessary. The most common radiographic finding on contrast-enhanced MRI brain is pachymeningeal thickening and enhancement, which is distinct from disease-related leptomeningeal enhancement and is thought to reflect compensatory engorgement of the dural veins following reduction in CSF volume [57]. Pituitary enlargement, subdural effusions or hematomas, rounding of the dural venous sinuses, brain sagging, and tonsillar herniation are other less common features of intracranial hypotension [58].

Conservative measures are sufficient to alleviate 90% percent of PDPH, generally resolving within 7–10 days of LP. These include maintenance of supine positioning, adequate hydration, caffeine, and oral analgesics. Acetaminophen, gabapentin, hydrocortisone, and theophylline have the highest quality evidence for this purpose [59]. The combination analgesic, butalbital-acetaminophen-caffeine, is commonly employed in clinical practice and is likely safe in the short term despite lack of high-quality data to support its use; however, patients should be cautioned on the risk of medication overuse headache and dependency with this agent due to butalbital [60].

For moderate-to-severe headaches that do not resolve with conservative measures, an epidural blood patch (EBP) is recommended [61]. EBP is a minor surgical procedure that involves the administration of autologous blood to the epidural space adjacent to the site of spinal puncture, thereby sealing the dural hole and preventing further CSF extravasation. Complete or partial improvement in symptoms is observed in 93% and 98% of patients requiring one or two EBPs, respectively [62, 63]. Postural headache relief occurs within minutes to hours of a successful EBP, though mild-moderate pressure at the procedure site is common.

#### Prevention

Patients with leptomeningeal metastases often require repeated lumbar punctures for pressure and treatment response assessments. History of a prior severe PDPH often incites anxiety amongst those who require a subsequent LP, so identification of efficacious prevention strategies is important. Post-procedural immobilization and intense hydration are common recommendations to reduce the onset or severity of PDPH, despite suggestion that neither intervention significantly impacts outcomes [64]. At least one non-comparative study has suggested that fluoroscopy-guided dural punctures are associated with a lower incidence of PDPH (2.2%) compared to the historical literature [65]. The size and type of spinal needle, however, does definitively influence the incidence of a post-LP headache. This effect is more pronounced for cutting as opposed to non-traumatic pencil-point needles [66]. Therefore, in a patient with a history of a prior PDPH, use of a smaller bore needle and/or fluoroscopy-guidance to minimize number of pass attempts is reasonable to lessen the severity of recurrent headache. Post-procedural supine positioning, pre- and post-procedural intravenous hydration, and prophylactic oral analgesics can be considered on a case-by-case basis. Ommaya reservoir placement for ease of CSF access, particularly amongst those who are additionally candidates for intrathecal chemotherapy, obviates the need for further lumbar punctures and offers a more permanent solution.

### Headache Secondary to Intrathecal Chemotherapy

#### Pathophysiology

Intrathecal chemotherapy involves the administration of chemotherapeutics directly into the CSF, via Ommaya reservoir or lumbar puncture, in effort to maximize CSF drug exposure while minimizing systemic toxicities. Numerous agents have been studied in patients with leptomeningeal metastases, including methotrexate, cytarabine, thiotepa, topotecan, and trastuzumab, with variable disease-control rates based on cancer type [67–75]. Drug distribution and clearance is mediated by CSF bulk flow and absorption into the venous vasculature; however, this may be influenced by individual drug pharmacokinetics, active transport mechanisms in the choroid plexus (i.e., P-glycoprotein), and impediments to normal CSF flow as seen with elevated ICP or space-occupying obstructions [76]. However, drug administration directly into the intrathecal space causes a chemical arachnoiditis in 30–60% of patients, consisting of headaches, nausea, vomiting, back pain, and fever [71, 77]. Liposomal drug formulations offer a longer CSF half-life and less frequent dose administration with the trade-ff of higher rates of chemical arachnoiditis and potentially permanent adverse events [68, 78, 79].

Headaches with intrathecal drug administration may arise by 2 mechanisms: hyper-acute headache due to alterations in intrathecal volume which generally abates shortly after procedure completion and acute headaches within 1–4 days due to chemical arachnoiditis.

#### Presentation

The International Headache Society defines headache secondary to intrathecal injection as one that is present in both the upright and recombinant position, occurring within 4 days and remitting within 14 days of drug administration, and associated with signs of meningeal irritation [13]. Patients may complain of accompanying neck stiffness, nausea, vomiting, photophobia, back pain, or fever. Chemical arachnoiditis tends to develop early within the drug administration schedule, oftentimes following the first dose, and then subsides with subsequent dose reductions and prophylactic strategies [80].

#### Diagnosis and Management

Clinical history and temporal relationship of headache with drug administration is generally sufficient to support a diagnosis of intrathecal drug-induced chemical arachnoiditis. CSF analysis may demonstrate a mild-to-marked rise in CSF leukocytes and/or an upward shift granulocytic cell populations [80]. Due to the low risk of iatrogenic bacterial infection introduced by intrathecal access, CSF bacterial cultures should be evaluated in patients with new onset intrathecal chemotherapy-related headaches and particularly in those with severe meningeal signs and fevers [80]. Patients who present with atypical features, including those with subacute-onset headaches > 4 days after drug administration and/or with a positional component, should undergo investigation for alternative headache causes. Increased ICP may develop in patients receiving intrathecal therapy, which may be drug-induced or due to progressive leptomeningeal metastases, and would serve as a contraindication to additional intrathecal drug administrations due to CSF blockage.

Oral or intravenous corticosteroids are first-line therapy in patients with headaches secondary to drug-induced arachnoiditis, the dose of which may be tailored to patient response. Patients with more severe chemical arachnoiditis might require intravenous steroids and empiric antibiotics pending negative CSF cultures.

#### Prevention

A history of prior chemical arachnoiditis does not necessarily preclude future doses of intrathecal chemotherapy. Steroid prophylaxis for 2–5 days surrounding intrathecal drug administration is generally effective in minimizing future occurrences, with or without additional supportive medications such as anti-emetics. Slower drug administration over 2–5 min helps to minimize intra-procedural head discomfort due to shifts in ventricular pressure. If severe chemical arachnoiditis recurs, then dose reduction or drug discontinuation may be necessary.

### Headache Secondary to Cranial Irradiation

#### Pathophysiology

Radiation therapy offers palliative treatment of symptomatic or nodular sites of leptomeningeal metastases, most commonly in the form of whole brain radiation therapy and/or focal spinal radiation, albeit with only modest impact on patient survival [81, 82]. More recently, proton craniospinal irradiation has emerged as an efficacious alternative to standard photon-beam involved-field radiation, offering comprehensive treatment to the entire neuraxis and consequently a survival benefit in select patients with adequate functional status [83••] Ionizing radiation exerts cancer cell death both directly via double- and single-stranded DNA breaks and indirectly via production of reactive oxygen and nitrogen species, followed by p53-mediated cellular apoptosis or senescence [84]. Healthy tissues are relatively radioresistant compared to cancer cells due to intact DNA repair pathways and low proliferation rate. However, in the setting of acute cranial radiation toxicity, resident microglia are activated both directly by ionizing radiation and indirectly through loss of resting anti-inflammatory cytokines from damaged neurons [85]. Activated microglia, together with astrocytes, release pro-inflammatory cytokines (TNFα, IL-1β, IL-6, IL-18), chemokines (CCL2, CXCL2), and reactive oxygen and nitrogen species, further perpetuating a state of neuroinflammation in the brain and CSF. Increased permeability of the blood-brain and blood-CSF barriers allows for penetration of T_H_2 lymphocytes, regulatory T cells, and M2 macrophages into the brain parenchyma, secreting anti-inflammatory factors in effort to dampen the acute inflammatory response and promote neuroregeneration.

#### Presentation

The combination of radiation-induced leptomeningeal cell apoptosis and neuro-inflammatory bystander effects on neuronal tissue is correlated with multiple neurologic symptoms in acute radiation toxicity. Headaches, fatigue, nausea, and vomiting occur in 10–20% of patients in the hours to days following cranial irradiation [86–88]. Symptoms may begin as early as the first fraction of radiation. Neurologic toxicity correlates with the burden of leptomeningeal disease and larger fields of radiation. In severe cases, neuroinflammatory-induced elevations in ICP can cause acute pressure crisis mid-treatment. Hyponatremia related to either syndrome of inappropriate anti-diuretic hormone secretion or cerebral salt wasting, both a consequence of neurologic injury, may develop during radiation therapy and amplify clinical deterioration [89].

#### Diagnosis and Management

Patients suffering from mild headaches and neurocognitive decline during treatment with cranial irradiation may be observed with corticosteroids and supportive measures. However, moderate-severe neurotoxicity requires prompt attention in order to prevent clinical deterioration during treatment. Since radiation toxicity causes headaches, nausea, and vomiting independent of ICP, a low threshold for head imaging and diagnostic lumbar puncture is warranted to differentiate acute radiation toxicity from rising ICP. Temporizing lumbar punctures every 48–72 h or ventriculoperitoneal shunt placement may be necessary to enable the patient to tolerate a complete course of cranial irradiation.

#### Prevention

Patients with a high burden of leptomeningeal disease or pre-existing headaches should be treated with a palliative dose of corticosteroids during their radiation course, to be tapered slowly once radiation has completed. Measurement of ICP prior to initiation of cranial irradiation should be strongly considered, particularly in any with positional symptoms, due to the high risk of pressure crisis with radiation treatments.

## Conclusion

Headache is a common but treatable symptom of leptomeningeal metastases with careful consideration of the underlying headache mechanism. Patients with leptomeningeal metastases can generally be subdivided into 5 non-mutually exclusive headache sub-classifications with varying pathophysiologies, including headaches secondary to disease-related meningeal irritation, progressively increased ICP, iatrogenic low ICP following dural puncture, intrathecal chemotherapy-induced chemical arachnoiditis, and cranial irradiation-induced neuroinflammation.

**Fig. 2:**
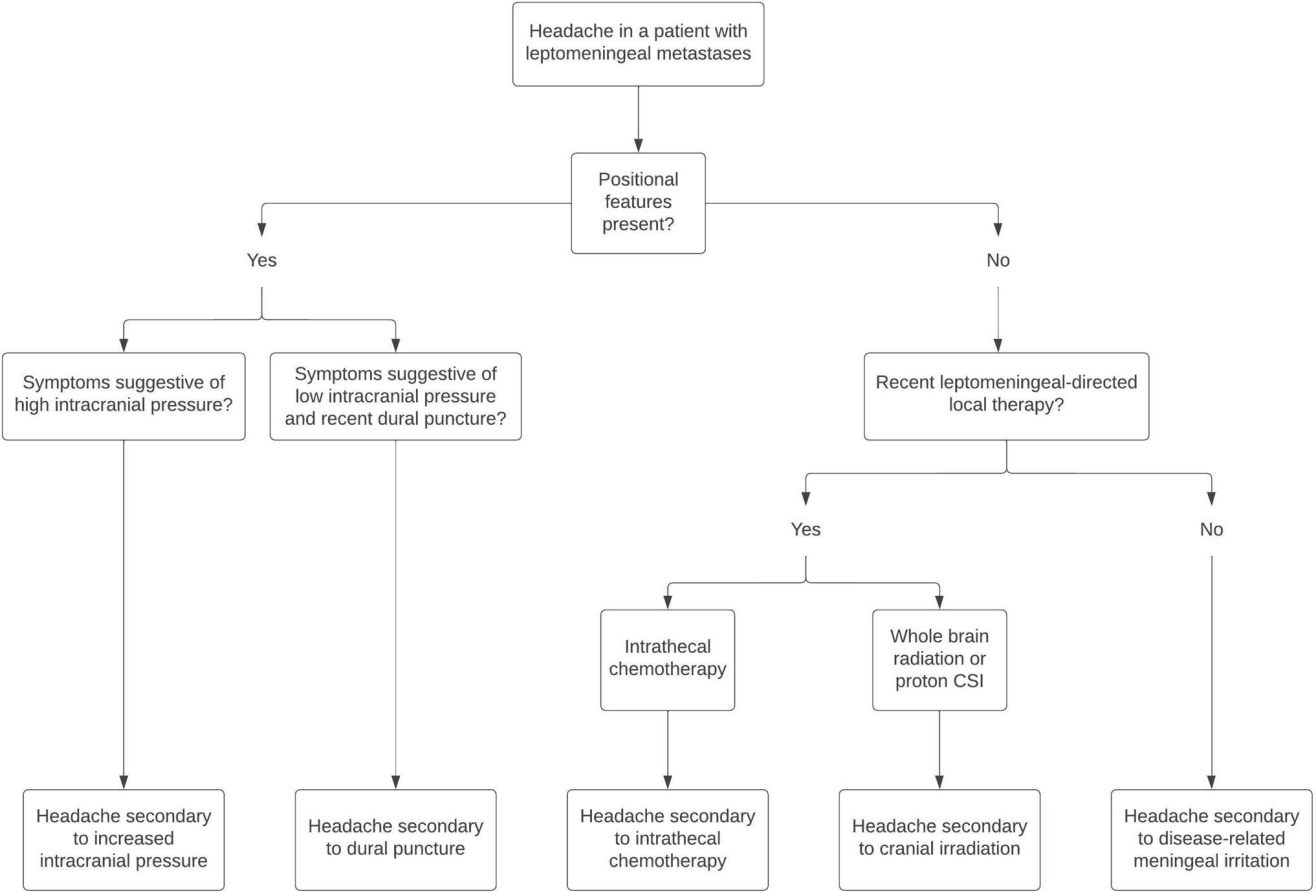
Clinical algorithm in differentiating headache subtypes in patients with leptomeningeal metastases. When assessing headaches in a patient with leptomeningeal metastases, inquiring about the presence of positional symptoms is an essential first step in the diagnosis of elevated intracranial pressure or intracranial hypotension, in the appropriate clinical setting. If pressure-mediated headache subtypes are sufficiently ruled out on clinical or diagnostic grounds, the clinician should then inquire about any recent leptomeningeal-directed cancer therapies, such as intrathecal chemotherapy or cranial irradiation, which can independently induce inflammation in the subarachnoid space. In the absence of these relevant details, headache secondary to potentially progressive leptomeningeal metastasis and the associated CSF inflammatory response is likely, with other non-cancer-related primary headache disorders, a diagnosis of exclusion

When approaching a patient with headaches and leptomeningeal metastases, the diagnostic algorithm (Fig. 2) should first consider the presence or absence of positional features (ie, headaches, nausea, vomiting, neck stiffness). Perturbations of ICP can occur anytime during a patient’s disease course, independent of leptomeningeal-directed treatments, and may have emergent therapeutic consequences. Rising ICP can manifest in both nebulous and acute presentations, and so, a low threshold for diagnostic lumbar puncture is essential to determine if permanent CSF diversion is either therapeutically indicated and/or within the patient’s goals of care. Alternatively, iatrogenic low ICP following a lumbar puncture should be managed conservatively for a period of generally 3–7 days before considering if an EBP is necessary. After ICP alterations have been sufficiently ruled out on clinical or diagnostic grounds, review of the presence or absence of recent local leptomeningeal-directed therapies can point the clinician towards generally steroid-responsive headache subtypes. When corticosteroids and additional supportive measures are insufficient to alleviate headaches, an alternative primary or secondary headache disorder as per ICDH-3 may be considered, but only as a diagnosis of exclusion. Careful integrated analysis of the patient’s clinical and treatment history, neurologic examination, and diagnostic studies is essential to accurately deduce the root cause of leptomeningeal metastasis-associated headache and tailor supportive treatment recommendations accordingly.

## Data Availability

No datasets were generated or analyzed during the current study.
